# Role of Acupuncture in the Management of Severe Acquired Brain Injuries (sABIs)

**DOI:** 10.1155/2018/8107508

**Published:** 2018-09-12

**Authors:** Loredana Cavalli, Lucia Briscese, Tiziana Cavalli, Paolo Andre, Maria Chiara Carboncini

**Affiliations:** ^1^Severe Acquired Brain Injuries Unit, Cisanello University Hospital, Pisa, Italy; ^2^Dipartimento Chirurgico Ortopedico, Ospedale Carlo Poma di Mantova, Italy; ^3^Dipartimento di Scienze Mediche, Chirurgiche e Neuroscienze, University of Siena, Italy

## Abstract

Acupuncture therapy has been used to treat several disorders in Asian countries and its use is increasing in Western countries as well. Current literature assessed the safety and efficacy of acupuncture in the acute management and rehabilitation of patients with neurologic disorders. In this paper, the role of acupuncture in the treatment of acute severe acquired brain injuries is described, acting on neuroinflammation, intracranial oedema, oxidative stress, and neuronal regeneration. Moreover, beneficial effects of acupuncture on subacute phase and chronic outcomes have been reported in controlling the imbalance of IGF-1 hormone and in decreasing spasticity, pain, and the incidence of neurovegetative crisis. Moreover, acupuncture may have a positive action on the arousal recovery. Further work is needed to understand the effects of specific acupoints on the brain. Allegedly concurrent neurophysiological measurements (e.g., EEG) may help in studying acupuncture-related changes in central nervous system activity and determining its potential as an add-on rehabilitative treatment for patients with consciousness disorders.

## 1. Introduction

Severe acquired brain injuries (sABIs) include a variety of acute brain lesions characterized by the occurrence of variably prolonged coma (24 hours), and simultaneous motor, sensory, cognitive, and/or behavioural impairment that causes a certain degree of disability. Congenital, perinatal onset, or degenerative-progressive brain injuries are excluded from this definition.

A common consequence of sABIs is disorders of consciousness (**DOCs**), a prolonged cognitive impairment including the loss of awareness of oneself and environment. DOCs represent one of the greatest challenges that modern medicine faces today, with a huge burden of care for families and facilities. On the basis of the current taxonomy of DOCs, a state of altered consciousness can be categorized into coma, vegetative state (VS), also referred to as unresponsive wakefulness syndrome (UWS), and minimally conscious state (MCS) [1].

The most common cause of sABI is traumatic brain injury (**TBI**), a major source of death and disability worldwide. Two further causes of sABI are anoxic encephalopathy (**AE**), usually due to cardiocirculatory arrest (secondary to extensive myocardial injury and/or malignant arrhythmias), and** ischemic or haemorrhagic stroke**. These conditions mostly affect subjects from the fifth decade onwards and represent about 40% of the sABIs. The main involvement of neurons leads to a worse prognosis than TBI. In AE, the neurons disruptions, with a low regenerative potential, cause a high risk of irreversibility of the consciousness disorder. Moreover, the time interval within which a recover is reasonable, up to 12 months from the event for TBI, is unlikely more than 3-6 months from the event for anoxic or vascular brain injuries. Late recoveries are possible, but rare. Further nontraumatic sABI arises from brain tumors, infections, and toxic-metabolic encephalopathy [2].

Despite the fact that steady progress has been made toward prolonging patients survival and several pharmacologic and neuromodulating strategies have been proposed, results on functional recovery of DOCs are still scarce.

## 2. Physiopathology of DOCs

The immediate effect of the impact forces on intracranial tissues, i.e., the** primary brain injury,** can be focal or diffuse. Focal injuries, such as contusion, laceration, and haemorrhage, are early detectable upon imaging and their consequences depend on their location and severity. Diffuse brain injuries, like concussion or diffuse axonal injury, require magnetic resonance to be detected [3]. However, what is susceptible of treatment is** secondary brain injury** (SBI), the cascade of biochemical and cellular events developing minutes to months after the insult: ionic imbalance (due to hypoxia and hypotension), glutamate excitotoxicity, oxidative stress generated by mitochondrial dysfunction, ischemic injury, edema formation, intracranial hypertension, neuroinflammation (by both systemic and central neuron system immunoactivation), and blood-brain barrier (BEE) disruption [3, 4], as schematized in Table 1.

Several secondary processes are based on intracellular** calcium** overload, due to excitatory amino acids and inflammatory cytokines released. In absence of oxygen, glucose enters the glycolytic pathway, where it is converted to NADH and pyruvate; the latter becomes lactic acid just producing 2 ATP molecules, while Krebs cycle is precluded. Thus, a downregulation of ATP-dependent Na^+^/K^+^-ATPase pump leads to sodium overload, which acting on Na^+^/Ca^2+^ exchange pump provides calcium retention.

Analogously, the huge release of glutamate following a head trauma, in hypoxic conditions, is not counterbalanced by astrocyte reuptake of glutamate, which acts on N-methyl-d-aspartate (NMDA) receptor, thus increasing calcium influx. Calcium is therefore sequestered by mitochondria causing their dysfunction, lysis, and release of byproducts [3]. Moreover, calcium stimulates neuronal and endothelial cells overproduction of nitric oxide (NO), which can be associated with oxidative stress. A further effect of calcium overload is mitochondrial-mediated apoptosis following the release of cytochrome c.

In response to primary brain injury, an** inflammatory response** is provided by microglia and astrocytes, which attract leukocytes via cytokines, chemokines, and NO. The disruption of the BEE often leads to cerebral oedema and intracranial hypertension [3].

## 3. Reactions to Brain Injury

A series of mechanisms are activated at neuronal (neurogenesis, synaptogenesis, and dendritic remodelling) glial, and vascular level, referred to as***neuroplasticity*** [5]. These events are promoted by increased expression of growth factors involved in brain development, such as nerve growth factor (NGF), neurotrophin 4/5, basic fibroblast growth factor, and brain derived neurotrophic factor (BDNF); an increasing importance has also emerged for insulin-like growth factor 1 (IGF-1), regulating metabolic function, oligodendrocyte proliferation, and survival, angiogenesis, myelinisation, and neurite outgrowth [5] and receptors for IGF-1, virtually ubiquitary, are mainly expressed by mesenchymal origin-derived cells in the hippocampus, parahippocampus, amygdala, cerebellum, and cortex [6].

## 4. Available Strategies to Manage sABIs

New insights into the pathophysiology of sABIs initiated new therapeutical approaches (neuroprotective strategies) aimed at interrupting secondary brain injury development and promoting mechanisms of arousal (see Table 2). Among the neuroprotective strategies, mild hypothermia (which decreases cerebral metabolic demand, excitatory neurotransmitter release, inflammation, and oedema), hyperosmolar therapies (also known for their immune-modulating property), statin (reducing oxidative stress and inflammation), and cyclosporin A (ameliorating mitochondrial function) have been proposed [3]. For arousal recovery, dopaminergic, serotoninergic and GABAergic drugs have been explored, with occasional results. Several nonpharmacological strategies have been utilized. Early verticalization has been shown to increase the spectral power of the EEG higher frequencies and the subject's arousal [7, 8], probably enhancing vestibular afference on locus coeruleus, raphe, and thalamic intralaminar nuclei. Enriched environment, equipped with emotional stimuli and biographically meaningful objects, showed greater range of behavioural responses [9]. Finally neuromodulatory techniques, including deep brain stimulation (DBS), transcranial direct current stimulation (TDCS), transcranial magnetic stimulation (TMS), spinal cord stimulation, median nerve stimulation, or vagus nerve stimulation, have been proposed.

## 5. A Complementary Approach for the sABIs' Management Derivating from Ancient Chinese Medicine:* Acupuncture*

The aim of this review is to explore the potential role of acupuncture (1) in the acute treatment of sABIs, in containing secondary brain injury by acting on different pathophysiologic mechanisms, and (2) in the subacute/chronic management of sABI outcomes, through a limitation of spasticity, pain, dysautonomia, and a possible action on the arousal recovery.

Acupuncture is a traditional medicine traced back to over 3000 years ago in China [10], consisting in inserting needles into specific points of the patient's body (“acupoints”) chosen on the basis of the Meridian theory of Traditional Chinese Medicine (TCM) [11]. It has been used in the treatment of stroke and its consequences for over 2,000 years in China.

Under the influence of neuroanatomy, neurophysiology, and bioholographic principle of modern medicine, in the early 1970s, scalp acupuncture, one of the several specialized acupuncture techniques in which a filiform needle is used to penetrate specific stimulation areas on the scalp mainly for the treatment of brain diseases, was set up and separated from traditional acupuncture system [12].

Scalp acupuncture has been reported to (1) improve cerebral blood circulation, promoting regional energy metabolism; (2) upregulate expression of glial cell-line derived neurotrophic factor (GDNF), possibly promoting proliferation and differentiation of neural stem cells in the focal cerebral cortex and hippocampus; (3) reduce contents of excitatory amino acid and increase level of GABA, thus lowering neurogenic toxicity; (4) ease cerebral vascular immunoinflammatory reactions; (5) regulate blood lipid metabolism to resist cerebral free radical damage; and (6) inhibit cerebral cortical apoptosis [13].

### 5.1. Acupuncture and Inflammation in DOCs

Few studies considered the diachronic assessment of inflammatory markers after a sABI in order to monitor the local and systemic stress response in DOCs. Amico and colleagues evaluated the subacute phase of DOCs in five patients in vegetative state (VS) and one in minimal consciousness state (MCS), by clinical assessment and biochemical analyses [14]. A positive correlation was found between the serum levels of** osteopontin** (OPN), a cytokine involved both in neuroinflammation and neurorepair, and prognosis, with the lowest level detected in the patient who then emerged from MCS, the highest in the one who then died. Moreover, the lymphocyte subset presented a general increase of CD4+/CD3+ ratio, with a suspect unbalance of CD4+ toward Th2; prolactin resulted to be the best endocrinological marker of sABI [14].

Applied at* Baihui* (**GV20**) and left* Zusanli* (**ST36**), acupuncture significantly reduced the infiltration of inflammatory cells and the expression of the proinflammatory enzyme MMP2 in cerebral ischemia/reperfusion injury (CIRI) model rats. In particular they attenuated the expression of the** water channel proteins P4 and AQP9** in the ischemic brain, leading to the mitigation of inflammation-related brain edema. Consistent with the smaller observed infarct size, acupuncture and EA both promoted significant improvements in the Modified Neurological Severity Score (mNSS) in CIRI model rats, indicative of enhanced neurological function [15].

Moreover, acupuncture successfully downregulates tumor necrosis factor alpha (TNF-*α*), which results in anti-inflammatory responses. The neural pathways by which acupuncture signalling stimulates anti-inflammatory effects have been mapped. By testing the effects that a splenic neurectomy and vagotomy have on TNF-*α* levels in the spleen and the brain, Lim et al. found that the anti-inflammatory effects of manual acupuncture at** ST36** rely on the vagus nerve pathway. Moreover, both manual acupuncture stimulation (MAC) and electroacupuncture (EA) induce c-Fos protein generation [16].

### 5.2. Acupuncture and Redox Equilibrium

Oxidative stress, the imbalance between the production of reactive oxygen and nitrogen species (ROS/RNS) and the endogenous antioxidant system, causing a cascade of chain reactions resulting in cellular damage, is a critical feature in the pathological process of various diseases [17].

Recently, a large body of evidence demonstrated that acupuncture has antioxidative effect in various conditions [18–20], although the exact mechanism, especially the influence of acupuncture on signalling pathways, remains unclear. Through redox system, antioxidant system, anti-inflammatory system, and nervous system, acupuncture could make the oxidative damage and the antioxidant defence remain in a relatively constant redox state. However, the recent acupuncture researches about oxidative stress are sporadic and preliminary [21].

### 5.3. Acupuncture and Intracellular Calcium

The insertion of the needle represents an effective mechanical stimulus, leading to tissue displacement and to intracellular calcium increase and signalling. The modulation of calcium channels seems to be the primary mechanism for endorphin secretion and release from immunocytes and for the inhibitory effects of opioids on peripheral neurons [22]. Thus,** calcium** ion, whose increase is so crucial in the pathogenesis of SBI, may be taken as the carrier of the biological modulation system provided by acupuncture, where the mechanical wave onsets an acoustic shear wave, and this drives to calcium signalling [22].

### 5.4. Acupuncture and Neuron Regeneration

In the brain of human adults, neural stem cells (NSCs) have been demonstrated in the pallium, subependymal region, hippocampus, and corpus striatum, which have the ability of self-duplication, self-regeneration, and differentiation into neurons and glial cells. During cerebral ischemia reperfusion, astrocytes play a crucial role in limiting neuronal lesion, as they release epoxyeicosanoic acids in order to enlarge brain vessels, release Nerve Growth Factors to make neurons survive and axons grow, produce neurotransmitters, metabolize toxic molecules, and have also the potentiality to become NSCs [23, 24].

The astrocyte activation and proliferation marker is Glial Fibrillary Acidic Protein (**GFAP**), while Neuron Specific Enolase (NSE) is one of the neurons' markers. Acupuncture on the conception (CV) and governor vessels (GV) has been shown to inhibit excessive proliferation of astrocytes and promote NSCs differentiation in the ischemic brain [25, 26]. In particular, needling acupoints** GV20** and** GV26** could downregulate the number of GFAP+ cells, while increasing the GFAP/NSE double–labelled cells in the hippocampal dentate gyrus [25, 26]. Another study, which employed a rat TBI model, proved that during the early post-TBI stage, acupuncture (**GV20, GV26, GV15, GV16, and LI4**) can promote the proliferation and differentiation of NSCs and glial cells, which is crucial to control neuronal necrosis; in the late phase, it can inhibit glial proliferation and differentiation, driving to neuron and oligodendrocytes regeneration and tissue repair [27].

Moreover, needling** CV24, CV4,** and** CV3** has been shown to upregulate the expression of basic FGF, EGF, and NGF after cerebral ischemia reperfusion, activating nerve repair and proliferation of neuronal precursors [28, 29].

As regards human studies, recent evaluations used single-photon emission computed tomography (SPECT) and T2-weighted imaging (T_2_WI) [30], or functional magnetic resonance imaging (fMRI) [31]. Shen and colleagues compared serial diffusion tensor imaging (DTI), fluid-attenuated inversion recovery (FLAIR), and T_2_WI performed in 20 patients with recent cerebral infarction in the basal ganglia, randomly divided into an acupuncture group and a control group [32]. The apparent diffusion coefficient (ADC) of infarction lesions, decreased at stroke onset, was showed significantly elevated after the acute stage, while the ADC of the bilateral cerebral peduncle was reduced on the infarction side. Fractional anisotropy (FA) values of abnormal signals on DTI in the infarction areas and cerebral peduncles underwent a significant reduction from stroke onset to the chronic stage. Interestingly, a significant difference in ADC and FA values between the two groups was observed, with a higher FA value in the acupuncture group than the control group, thus suggesting the effectiveness of acupuncture for** protecting neurons by postponing Wallerian degeneration** of brain infarction, and facilitating recovery [32].

### 5.5. Acupuncture and Arousal

The pathology of** disorders of consciousness** can be represented by (A) damage of Reticular Ascending System (B) large-scale damage to cerebral cortex, (C) injury to links (e.g., thalamus) between cerebral cortex and brain stem, and (D) injury to connections (e.g., corpus callosum) within the cerebral cortex, i.e., severe diffuse axonal injury (DAI).

The production of inhibitors (including GABA) induced by brain injury generates a response resembling automatic shutdown, probably aimed at conserving energy and promoting cell survival, but causing a comatose state [33]. Therefore, any treatment affecting the reticular activating system may be worth trying, and, among the possible treatments, acupuncture has the highest potential [34].

Recent studies on resting state (RS) in DOC, by using functional magnetic resonance imaging (fMRI), showed that functional connectivity is severely impaired above all in the Default Mode Network (DMN). In the vegetative and minimally conscious state, DMN integrity seems to correlate with the level of remaining consciousness.

The DMN is among the most robust networks found with resting state fMRI and encompasses the posterior cingulate cortex (PCC)/precuneus, mediofrontal/anterior cingulate cortex, and temporoparietal cortex [35]. Activity in the DMN diminishes when the brain is involved in attention-demanding cognitive tasks [36] and returns to its prominent presence when no such task is being performed.

The DMN seems to be of particular interest, as its connectivity has been shown to decrease during loss of consciousness, and PET studies have shown an increase in neuronal activity in DMN regions (especially in the PCC/precuneus) upon recovery from VS [37]. Indeed, Vanhaudenhuyse [38] and Fernández-Espejo [39] et al. observed a correlation between the DMN integrity and the level of consciousness in noncommunicative brain-damaged patients.

Imaging evidence has been provided to support that electroacupuncture at** GV20**, employed to treat major depressive disorders, may modulate the Default Mode Network (**DMN)**, the cerebral functional network encompassing the posterior and anterior cortical midline structures, which is considered to be involved in stimulus-independent thought, mind-wandering, and self-consciousness. EA at** GV20** would increase functional connectivity (FC) between the precuneus/posterior cingulate cortex (PC/PCC) and bilateral anterior cingulate cortex (ACC) and reduce FC between the PC/PCC and left middle prefrontal cortex, left angular gyrus, and bilateral hippocampus/parahippocampus [40]. These findings are of particular importance when considering DOCs, where resting state network activity reveals reduced interhemispheric connectivity and correlates with levels of consciousness.

The acupoint* Shuigou* (**GV26**), placed at the junction of the upper one-third and lower two-thirds of the philtrum midline, also has been described as promoting the function of GV meridian, closely related to brain function, decreasing cognitive impairment, and promoting neurogenesis in the APP/PS1 transgenic mice [41].

Interestingly, enhanced bodily attention can be triggered by genuine acupuncture at** PC6** and** HT7** acupoints, which were exhibited to activate the salience network (insula, ACC, secondary somatosensory cortex, and superior parietal cortex) and deactivate the DMN (medial prefrontal cortex, PCC, inferior parietal cortex, and parahippocampus) [42].

Combined with western medicine, electroacupuncture therapy at* Baihui* (**GV20**),* Shuigou* (**GV26**), and* Yongquan* (**KI1**), resulted effective in improving consciousness recovery of patients in coma due to TBI, both reducing awake time and increasing awake rate, compared to a control group receiving only western treatments [43].

#### 5.5.1. Autonomic Dysfunction in sABI

Autonomic nervous system (ANS) deregulation and/or dysautonomia is another severe consequence of brain injury, not well cleared. Dysautonomia affects in particular ninety percent of TBI patients during the first week, leading to sleep and heart rhythm disorders, and increasing specific biomarkers of neural damage [6]. Clinically, patients suffering from DOC can show the so called “paroxysmal sympathetic hyperactivity” (PSH), episodic sudden increase in vital signs, particularly heart rate, blood pressure, respiratory rate, and temperature, with possible diaphoresis (i.e., excessive sweating) and abnormal, unintentional movements, spontaneously or in response to external painful stimuli [44, 45]. A growing body of evidence suggests that ANS may mediate large-scale brain activation, in an extreme attempt to preserve body system homeostasis and regain consciousness [46]. The primary and secondary brain lesions have the potential to compromise both cortical and subcortical control mechanisms of the ANS.

Most often, TBI leads to sustained sympathetic activation, contributing to the high morbidity, with oxidative stress in the ANS and activated hypothalamic-pituitary-adrenal axis and hypothalamic-sympathoadrenal medullary axis [4].

### 5.6. Acupuncture and ANS Modulation

An increasing clinical evidence demonstrates that acupuncture is helpful in treating ANS dysfunctions, such as nausea and vomiting [47]. For example, acupoints stimulation has been shown to change the sympathovagal balance toward vagal predominance [48, 49].

Abnormalities in the ANS, such as sympathetic overactivation and/or parasympathetic hypoactivation, may generate and sustain chronic pain [50, 51].

Acupuncture at certain points could reduce sympathetic nervous system activity associated with pain [52] or during mental stress in patients with heart failure [53]. However, the neurobiological basis of these effects is not yet clear [54]. In order to explore the regulative effect on ANS by acupuncture, Sakatani and colleagues monitored** heart rate** by placing photoelectrical sensor on the first finger of eighteen healthy male adults, and thus low frequency (LF) amplitude (0.04–0.15 Hz) and high frequency (HF) amplitude (0.15–0.4 Hz) were calculated by power spectral analysis [54]. Real acupuncture performed at point Large Intestine 4 (LI4) of the right hand (r-LI4) was shown to determine significant decreases of HR and LF/HF and a significant increase of HF, indicating a parasympathetic activation as well as sympathetic depression [55].

Moreover, vagus nerve stimulation (VNS) increases metabolism in the forebrain, thalamus, and reticular formation [56]. It also enhances neuronal firing in the locus coeruleus, which leads to massive release of norepinephrine in the thalamus and hippocampus, a noradrenergic pathway essential for arousal, alertness, and the fight-or-flight response [57]. Recently, based on this rationale, Transcutaneous Auricular VNS (**taVNS),** a noninvasive stimulation developed for treating epilepsy and depression without the surgery-related risks [58, 59], was firstly employed by Yu-tian Yu and colleagues on a patient in vegetative state [60]. A further case with implanted VNS recovered behavioural responsiveness and enhanced brain connectivity patterns [61].

### 5.7. Acupuncture against Neuroendocrine Dysfunction

An imbalance of the pituitary and hypothalamus hormones and their axes is often associated with sABI, due to compression, edema, skull base fracture, haemorrhage, intracranial hypertension, or hypoxia. In the acute phase of sABI, low IGF-1 with elevated GH levels have been detected, with increasing IGF-1 and normalizing GH concentrations in the following weeks. The IGF-1 upregulation and the disruption of BEE that persists until 7 days after injury, allowing a wide level of hormone to reach the surviving neurons, probably play a role in promoting neurite overgrowth, inducing progenitor cell differentiation and inhibiting neuron apoptosis [6].

In rat model of renal failure- (RF-) induced hypertension, stimulation with acupuncture, and most significantly with EA, at** ST36** and** KI3**, not only attenuated glomerulosclerosis and tubulointerstitial fibrosis, but corrected the decreases in RF-induced IGF-1 mRNA and protein levels, thus counteracting oxidative stress [62]. These finding may suggest the ability of acupuncture in restoring IGF-1 function in any situations where its levels are reduced, including sABI, although no studies have been conducted on this purpose.

#### 5.7.1. Pain and DOC

The experience of pain in disorders of consciousness is still debated. Neuroimaging studies, using functional magnetic resonance imaging (fMRI) [63, 64], Positron Emission Tomography (PET) [65], multichannel electroencephalography (EEG), and laser-evoked potentials [66], suggest that the perception of pain increases with the level of consciousness.

VS and MCS are by definition incompatible with a reliable and consistent ability to communicate about pain experiences, while the nature of these conditions is characterized by various factors that can give rise to pain (e.g., spasticity, contractures, etc.) [67].

### 5.8. Acupuncture and Pain Relief

Many different mechanisms may explain the analgesic effect of acupuncture. Among these is the***gate control theory*** of pain proposed by Melzack and Wall in 1965 [68]. Specific nerve fibers would transmit pain to the spinal cord, while other nerve fibers inhibit pain transmission. Both groups of fibers met at the* substantia gelatinosa* in the spinal cord, where pain and pain inhibitory stimuli were integrated. Pain would be perceived only if the noxious input exceeded the inhibition of pain. However, gate control theory cannot explain the full spectrum of acupuncture effects, and in particular the prolonged pain relief.

Since the 1970s, the secretion of a range of biochemicals or neurotransmitters has been considered among the mechanism of acupuncture analgesia, such as** adenosine, opioid peptides, cholecystokinin octapeptide, 5-hydroxytryptamine, noradrenalin, glutamate, GABA, substance P, calcium ions, angiotensin II, somatostatin, arginine vasopressin, and dopamine** [69–81].

Different subtypes of opioid receptor were also believed to mediate the frequency-dependent electroacupuncture analgesia [82, 83]. For example, EA at a low frequency of 2 Hz would facilitate the release of enkephalin, but not dynorphin, while a high frequency of 100 Hz would stimulate the dynorphin but not enkephalin release in rats [82], as well as in humans [83]. The primary foundations for acupuncture effects seem to be** bioelectromagnetic**, while biochemical factors would be secondary. By the way, the opioid peptide secretion was recently proposed as being due to mechanical acoustic shear wave activation and calcium signalling induced by** needle rotation **[22].

Recently, the inflammatory reflex (via the ANS) has been observed as potentially crucial for the antihyperalgesic effect of acupuncture: by regulating the immune system it can elucidate not only the analgesic, but also the anti-inflammatory mechanism of acupuncture [76].

### 5.9. Acupuncture and Control of Spasticity

Spasticity is a frequent consequence of sABIs, arising from an anarchic reorganization of the pyramidal and parapyramidal fibers, and leading to hypertonia and hyperreflexia of the affected muscular groups and, if untreated, to possible irreversible joint lesions. Current treatment options include intrathecal baclofen and soft splints, botulinum toxin, or cortical activation by thalamic stimulation [84]. There is a low quality evidence for rehabilitation programs, extracorporeal shock-wave therapy, transcranial direct current stimulation, transcranial magnetic stimulation, and transcutaneous electrical nerve stimulation targeting spasticity, while a moderate evidence has been shown for electroneuromuscular stimulation and acupuncture as an adjunct therapy to conventional routine care (pharmacological and rehabilitation) [85]. In patients with DOCs, acupuncture at** GV26, Ex-HN3, LI4, and ST36 **was proven to reduce spastic muscle hypertonia by decreasing the excitability of the spinal motor neurons, both ten minutes after needles insertion and ten minutes after their removal [86].

## 6. Final Considerations and Conclusions

The World Health Organization has recommended acupuncture in 1980 as an effective complementary therapy for several diseases. Among the indications, neurologic disorders have been shown to benefit from acupuncture.

In this review, we analysed scientific studies and clinical reports that explored the acupuncture's effects in several acquired brain injuries, aiming to

(1) limit brain secondary injury, by acting on systemic and local inflammation, oxidative stress, intracellular calcium overload, neuron regeneration, and growth factors release;

(2) manage sABI consequences, such as neuroendocrine and autonomic dysfunction, muscle spasticity, and pain.

Research in this field has obtained significant improvement with the technical support of the life sciences, and the studies of acupuncture have in turn accelerated the development of biomedical science. However, intrinsic aspects of this medical approach make it difficult to run a clinical study, and several data derive from animal studies or from small-size and heterogeneous samples of patients. Moreover, the acupoints selected for treating sABI can differ between research groups.

In addition, patients with disorders of consciousness are* per se* difficult to study. Given the impossibility of communicating with the patient, the content of consciousness can be only inferred by response behaviour. Diagnostic errors may depend not only on the operator but also on wakefulness fluctuations of the patient, who may be drowsy or agitated, or have epileptic seizures or aphasia. The neurophysiologic evaluations are made difficult by the presence of sweat of the head (which worsens the EEG impedance), muscle hypertonus (that obstructs mobilization), and noise from electromedical equipment, while functional imaging techniques are expansive and not easily available.

Further studies are needed to identify the most efficient and customized therapeutical protocol, aiming in particular at eliciting arousal.

**Figure 1:**
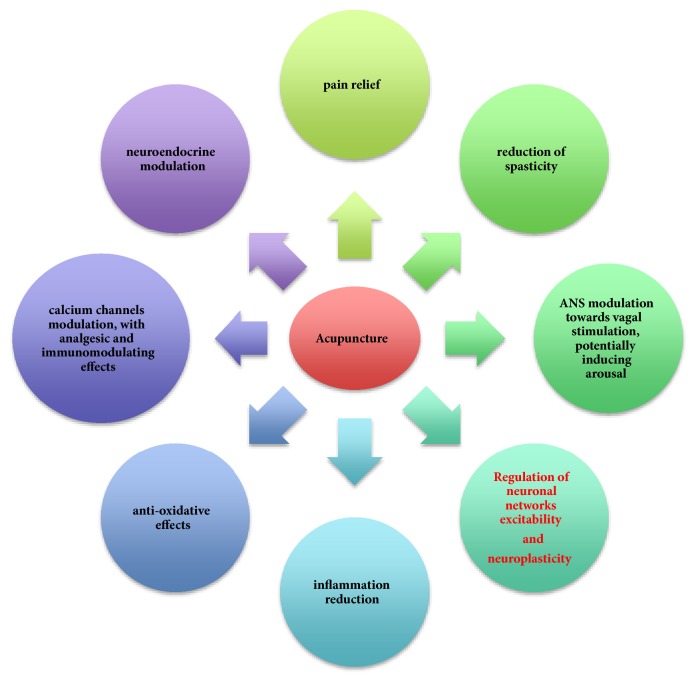
Role of acupuncture in the management of sABIs.

The available data suggest that, in patients with sABIs/DOCs, acupuncture may represent an interesting frontier in the years ahead, as it seems to limit the secondary brain injury development, modulate ANS, and ameliorate their quality of life (Table 3, Figure 1). The absence of side effects or drug interactions make it particularly indicated for such fragile subjects.

## Figures and Tables

**Table 1:** Physiopathology of DOCs.

Primary brain injuries	secondary brain injuries
**Focal**	
(i) contusion	(i) ionic imbalance (due to hypoxia and hypotension)
(ii) laceration	(ii) glutamate excitotoxicity
(iii) hemorrhage	(iii) oxidative stress (generated by mitochondrial dysfunction)
**Diffuse**	
(i) concussion	(iv) ischemic injury
(ii) diffuse axonal injury	(v) edema formation
	(vi) intracranial hypertension
	(vii) neuroinflammation
	(viii) blood-brain barrier disruption

**Table 2:** Available strategies to manage sABIs and their mechanisms of action.

**NEUROPROTECTIVE STRATEGIES**	

Mild hypothermia	*decrease of cerebral metabolic demand, excitatory neurotransmitter release, inflammation and edema*

hyperosmolar therapies	*immune-modulating effects*

statins	*reducing oxidative stress and inflammation*

cyclosporin A	*ameliorating mitochondrial function*

**NEUROMODULATIVE STRATEGIES**	

**Pharmacologic** (i) dopaminergics (ii) serotoninergics (iii) gabaergics	*neurotransmitter modulation*

**Non Pharmacologic** postural verticalization	*potential enhancement of the vestibular effects on locus coeruleus, raphe and thalamic intralaminar nuclei*

**Neuromodulative Techniques:** (i) deep brain stimulation (DBS) (ii) transcranial direct current stimulation (TDCS) (iii) transcranial magnetic stimulation (TMS) (iv) spinal cord stimulation (v) median nerve stimulation (vi) vagus nerve stimulation	*increasing metabolism in the forebrain, thalamus and reticular formation.* *modulating neuronal networks and ANS*

**Table 3:** Acupuncture and neuroplasticity.

**MAIN MECHANISMS BY WHICH ACUPUNCTURE MAY INFLUENCE THE PHYSIOLOGIC PLASTIC REACTIONS TO BRAIN INJURY**	

*(i) Inhibition of brain neuronal apoptosis*	*(vii) Reduction of blood-brain barrier permeability in intracerebral haemorrhage (caveolin-1/matrix metalloproteinase)*

*(ii) Inhibition of aberrant astrocyte activation*	*(viii) Regulation of blood lipid metabolism to counteract cerebral free radical damage*

*(iii) Upregulation of neurotrophins expression*	*(ix) Promoting cerebral vascular immunoinflammatory reactions*

*(iv) Upregulate expression of GDNF *	*(x) Increase of GABA level*

*(v) Increased functional connectivity*	*(xi) Reduce contents of excitatory amino acids*

*(vi) Enhanced neuroblast proliferation and differentiation *	

## References

[1] Giacino J. T., Ashwal S., Childs N. (2002). The minimally conscious state: definition and diagnostic criteria. *Neurology*.

[2] De Tanti A., Zampolini M., Pregno S., CC3 Group (2015). Recommendations for clinical practice and research in severe brain injury in intensive rehabilitation: the Italian Consensus Conference. *European Journal of Physical and Rehabilitation Medicine*.

[3] Tran L. V. (2014). Understanding the pathophysiology of traumatic brain injury and the mechanisms of action of neuroprotective interventions. *Journal of Trauma Nursing*.

[4] Toklu H., Tümer N. (2015). Oxidative stress, brain edema, blood–brain barrier permeability, and autonomic dysfunction from traumatic brain injury. *Brain Neurotrauma*.

[5] Madathil S., Saatman K. (2015). IGF-1/IGF-R signaling in traumatic brain injury: impact on cell survival, neurogenesis, and behavioral outcome. *Brain Neurotrauma*.

[6] Mangiola A., Vigo V., Anile C., De Bonis P., Marziali G., Lofrese G. (2015). Role and importance of IGF-1 in traumatic brain injuries. *BioMed Research International*.

[7] Elliott L., Coleman M., Shiel A. (2005). Effect of posture on levels of arousal and awareness in vegetative and minimally conscious state patients: a preliminary investigation. *Journal of Neurology, Neurosurgery & Psychiatry*.

[8] Greco A., Carboncini M. C., Virgillito A., Lanata A., Valenza G., Scilingo E. P. Quantitative EEG analysis in minimally conscious state patients during postural changes.

[9] Di Stefano C., Cortesi A., Masotti S., Simoncini L., Piperno R. (2012). Increased behavioural responsiveness with complex stimulation in VS and MCS: Preliminary results. *Brain Injury*.

[10] Han J.-S., Ho Y.-S. (2011). Global trends and performances of acupuncture research. *Neuroscience & Biobehavioral Reviews*.

[11] Xu B. R. (1986). *Clinical Acupuncture*.

[12] Liu Z., Guan L., Wang Y., Xie C.-L., Lin X.-M., Zheng G.-Q. (2012). History and mechanism for treatment of intracerebral hemorrhage with scalp acupuncture. *Evidence-Based Complementary and Alternative Medicine*.

[13] Tian L., Du X., Wang J. (2016). Scalp acupuncture twisting manipulation for treatment of hemiplegia after acute ischemic stroke in patients: study protocol for a randomized, parallel, controlled, single-blind trial. *Asia Pacific Clinical and Translational Nervous System Diseases*.

[14] Amico A. P., Annamaria T., Marisa M., Gianfranco M., Sabino D. (2013). Immune endocrinological evaluation in patients with severe vascular acquired brain injuries: Therapeutical approaches. *Endocrine, Metabolic & Immune Disorders—Drug Targets*.

[15] Xu H., Zhang Y., Sun H., Chen S., Wang F. (2014). Effects of acupuncture at gv20 and st36 on the expression of matrix metalloproteinase 2, aquaporin 4, and aquaporin 9 in rats subjected to cerebral ischemia/reperfusion injury. *PLoS ONE*.

[16] Lim H.-D., Kim M.-H., Lee C.-Y., Namgung U. (2016). Anti-inflammatory effects of acupuncture stimulation via the vagus nerve. *PLoS ONE*.

[17] Thomas S. R., Witting P. K., Drummond G. R. (2008). Redox control of endothelial function and dysfunction: molecular mechanisms and therapeutic opportunities. *Antioxidants & Redox Signaling*.

[18] Wang T., Liu C. Z., Yu J. C., Jiang W., Han J. X. (2009). Acupuncture protected cerebral multi-infarction rats from memory impairment by regulating the expression of apoptosis related genes Bcl-2 and Bax in hippocampus. *Physiology & Behavior*.

[19] Shi G., Liu C., Li Q., Zhu H., Wang L. (2012). Influence of acupuncture on cognitive function and markers of oxidative DNA damage in patients with vascular dementia. *Journal of Traditional Chinese Medicine*.

[20] Liu C., Yu J., Zhang X., Fu W., Wang T., Han J. (2006). Acupuncture prevents cognitive deficits and oxidative stress in cerebral multi-infarction rats. *Neuroscience Letters*.

[21] Zeng X.-H., Li Q.-Q., Xu Q., Li F., Liu C.-Z. (2014). Acupuncture mechanism and redox equilibrium. *Evidence-Based Complementary and Alternative Medicine*.

[22] Yang E. S., Li P.-W., Nilius B., Li G. (2011). Ancient Chinese medicine and mechanistic evidence of acupuncture physiology. *European Journal of Physiology*.

[23] Seri B., García-Verdugo J. M., McEwen B. S., Alvarez-Buylla A. (2001). Astrocytes give rise to new neurons in the adult mammalian hippocampus. *The Journal of Neuroscience*.

[24] Yang Z.-X., Chen P.-D., Yu H.-B. (2012). Research advances in treatment of cerebral ischemic injury by acupuncture of conception and governor vessels to promote nerve regeneration. *Journal of Chinese Integrative Medicine*.

[25] Yang Z. X., Yu H. B., Luo W. S. (2008). The effect of electroacupuncture at Ren and Du Vessels on hippocamp horizontal cells of focal cerebral ischemia. *China Medical Herald*.

[26] Yang Z., Yu H., Rao X., Liu Y., Pi M. (2008). Effects of electroacupuncture at the conception vessel on proliferation and differentiation of nerve stem cells in the inferior zone of the lateral ventricle in cerebral ischemia rats. *Journal of Traditional Chinese Medicine*.

[27] Jiang S., Chen W., Zhang Y. (2016). Acupuncture induces the proliferation and differentiation of endogenous neural stem cells in rats with traumatic brain injury. *Evidence-Based Complementary and Alternative Medicine*.

[28] Yang Z. X., Ma X. M., Yu H. B. (2009). Expression of growth factor after local cerebral ischemia-reperfusion in rats and effects of electroacupuncturing Ren Vessel on it. *Chinese Archives of Traditional Chinese Medicine*.

[29] Ma X. M., Yang Z. X., Yu H. B. (2011). Effects of electroacupuncturing Ren Vessels on expression of IGF after focal cerebral ischemia-reperfusion in rats. *Chinese Archives of Traditional Chinese Medicine*.

[30] Lee J. D., Chon J. S., Jeong H. K. (2003). The cerebrovascular response to traditional acupuncture after stroke. *Neuroradiology*.

[31] Schockert T., Schnitker R., Boroojerdi B. (2010). Cortical activation by Yamamoto new scalp acupuncture in the treatment of patients with a stroke: A sham-controlled study using functional MRI. *Acupuncture in Medicine*.

[32] Shen Y., Li M., Wei R., Lou M. (2012). Effect of acupuncture therapy for postponing wallerian degeneration of cerebral infarction as shown by diffusion tensor imaging. *The Journal of Alternative and Complementary Medicine*.

[33] Clauss R., Nel W. (2006). Drug induced arousal from the permanent vegetative state. *NeuroRehabilitation*.

[34] Hu W. L., Hung Y. C., Chang C. H. (2011). Acupuncture for disorders of consciousness—a case series and review, acupuncture. *Clinical Practice, Particular Techniques and Special Issues, Marcelo Saad (Ed.)*.

[35] Mason M. F., Norton M. I., van Horn J. D., Wegner D. M., Grafton S. T., Macrae C. N. (2007). Wandering minds: the default network and stimulus-independent thought. *Science*.

[36] Raichle M. E., MacLeod A. M., Snyder A. Z., Powers W. J., Gusnard D. A., Shulman G. L. (2001). A default mode of brain function. *Proceedings of the National Acadamy of Sciences of the United States of America*.

[37] Laureys S., Boly M., Maquet P. (2006). Tracking the recovery of consciousness from coma. *The Journal of Clinical Investigation*.

[38] Vanhaudenhuyse A., Noirhomme Q., Tshibanda L. J.-F. (2009). Default network connectivity reflects the level of consciousness in non-communicative brain-damaged patients. *Brain*.

[39] Fernández-Espejo D., Junque C., Cruse D. (2010). Combination of diffusion tensor and functional magnetic resonance imaging during recovery from the vegetative state. *BMC Neurology*.

[40] Deng D., Liao H., Duan G. (2016). Modulation of the default mode network in first-episode, drug-naïve major depressive disorder via acupuncture at Baihui (GV20) Acupoint. *Frontiers in Human Neuroscience*.

[41] Cao J., Tang Y., Li Y., Gao K., Shi X., Li Z. (2017). Behavioral changes and hippocampus glucose metabolism in APP/PS1 transgenic mice via electro-acupuncture at governor vessel acupoints. *Frontiers in Aging Neuroscience*.

[42] Jung W.-M., Lee I.-S., Wallraven C., Ryu Y.-H., Park H.-J., Chae Y. (2015). Cortical activation patterns of bodily attention triggered by acupuncture stimulation. *Scientific Reports*.

[43] Liu J.-P., Yang Z.-L., Wang M.-S., Shi R., Zhu B.-P. (2010). Observation on therapeutic effect of electroacupuncture therapy for promoting consciousness of patients with coma. *Chinese Acupuncture & Moxibustion*.

[44] Chatelle C., Vanhaudenhuyse A., Mergam A. N. (2008). Pain assessment in non-communicative patients. *Revue Médicale de Liège*.

[45] Perkes I., Baguley I. J., Nott M. T., Menon D. K. (2010). A review of paroxysmal sympathetic hyperactivity after acquired brain injury. *Annals of Neurology*.

[46] Takahashi C., Hinson H. E., Baguley I. J. (2015). Autonomic dysfunction syndromes after acute brain injury. *Handbook of Clinical Neurology*.

[47] Streitberger K., Ezzo J., Schneider A. (2006). Acupuncture for nausea and vomiting: An update of clinical and experimental studies. *Autonomic Neuroscience: Basic and Clinical*.

[48] Huang S.-T., Chen G.-Y., Lo H.-M., Lin J.-G., Lee Y.-S., Kuo C.-D. (2005). Increase in the vagal modulation by acupuncture at Neiguan point in the healthy subjects. *American Journal of Chinese Medicine*.

[49] Nishijo K., Mori H., Yosikawa K., Yazawa K. (1997). Decreased heart rate by acupuncture stimulation in humans via facilitation of cardiac vagal activity and suppression of cardiac sympathetic nerve. *Neuroscience Letters*.

[50] Passatore M., Roatta S. (2006). Influence of sympathetic nervous system on sensorimotor function: whiplash associated disorders (WAD) as a model. *European Journal of Applied Physiology*.

[51] Schott G. D., Mathias C. J., Bannister S. R. (1999). Pain and the sympathyetic nervous system. *Autonomic Failure Oxford*.

[52] Arai Y.-C. P., Ushida T., Osuga T. (2008). The effect of acupressure at the extra 1 point on subjective and autonomic responses to needle insertion. *Anesthesia & Analgesia*.

[53] Middlekauff H. R., Hui K., Yu J. L. (2002). Acupuncture inhibits sympathetic activation during mental stress in advanced heart failure patients. *Journal of Cardiac Failure*.

[54] Sakatani K., Kitagawa T., Aoyama N., Sasaki M. (2010). Effects of acupuncture on autonomic nervous function and prefrontal cortex activity. *Advances in Experimental Medicine and Biology*.

[55] Streitberger K., Steppan J., Maier C., Hill H., Backs J., Plaschke K. (2008). Effects of verum acupuncture compared to placebo acupuncture on quantitative EEG and heart rate variability in healthy volunteers. *The Journal of Alternative and Complementary Medicine*.

[56] Henry T. R., Votaw J. R., Pennell P. B. (1999). Acute blood flow changes and efficacy of vagus nerve stimulation in partial epilepsy. *Neurology*.

[57] Dorr A. E. (2006). Effect of vagus nerve stimulation on serotonergic and noradrenergic transmission. *The Journal of Pharmacology and Experimental Therapeutics*.

[58] Fang J. L., Rong P. J., Hong Y. (2015). Transcutaneous vagus nerve stimulation modulates default mode network in major depressive disorder. *Biological Psychiatry*.

[59] Rong P., Liu A., Zhang J. (2014). Transcutaneous nerve stimulation for refractory epilepsy: a randomized controlled trial. *Clinical Science*.

[60] Yu Y.-T., Yang Y., Wang L.-B. (2017). Transcutaneous auricular vagus nerve stimulation in disorders of consciousness monitored by fMRI: The first case report. *Brain Stimulation*.

[61] Corazzol M., Lio G., Lefevre A. (2017). Restoring consciousness with vagus nerve stimulation. *Current Biology*.

[62] Oh Y., Yang E. J., Choi S., Kang C. (2013). The effect of electroacupuncture on insulin-like growth factor-I and oxidative stress in an animal model of renal failure-induced hypertension. *Kidney and Blood Pressure Research*.

[63] Zanatta P., Messerotti Benvenuti S., Baldanzi F. (2012). Pain-related somatosensory evoked potentials and functional brain magnetic resonance in the evaluation of neurologic recovery after cardiac arrest: A case study of three patients. *Scandinavian Journal of Trauma, Resuscitation and Emergency Medicine*.

[64] Markl A., Yu T., Vogel D., Müller F., Kotchoubey B., Lang S. (2013). Brain processing of pain in patients with unresponsive wakefulness syndrome. *Brain and Behavior*.

[65] Laureys S., Faymonville M. E., Peigneux P. (2002). Cortical processing of noxious somatosensory stimuli in the persistent vegetative state. *NeuroImage*.

[66] De Tommaso M., Navarro J., Ricci K. (2013). Pain in prolonged disorders of consciousness: Laser evoked potentials findings in patients with vegetative and minimally conscious states. *Brain Injury*.

[67] Thibaut A., Chatelle C., Wannez S. (2015). Spasticity in disorders of consciousness: A behavioral study. *European Journal of Physical and Rehabilitation Medicine*.

[68] Melzack R. (1976). Acupuncture and pain mechanisms. *Anaesthesist*.

[69] Sims J. (1997). The mechanism of acupuncture analgesia: a review. *Complementary Therapies in Medicine*.

[70] Staud R., Price D. D. (2006). Mechanisms of acupuncture analgesia for clinical and experimental pain. *Expert Review of Neurotherapeutics*.

[71] Ulett G. A., Han S., Han J.-S. (1998). Electroacupuncture: mechanisms and clinical application. *Biological Psychiatry*.

[72] Zhao Z.-Q. (2008). Neural mechanism underlying acupuncture analgesia. *Progress in Neurobiology*.

[73] Irnich D., Beyer A. (2002). Neurobiological mechanisms of acupuncture analgesia. *Der Schmerz*.

[74] Kong J J., Gollub R R., Huang T. T. (2007). Acupuncture de qi, from qualitative history to quantitative measurement. *The Journal of Alternative and Complementary Medicine*.

[75] Sun J., Zhu Y., Yang Y. (2013). What is the *de*-*qi*-related pattern of BOLD responses? A review of acupuncture studies in fMRI. *Evidence-Based Complementary and Alternative Medicine*.

[76] Lin J.-G., Chen W.-L. (2008). Acupuncture analgesia: a review of its mechanisms of actions. *American Journal of Chinese Medicine*.

[77] Goldman N., Chen M., Fujita T. (2010). Adenosine A1 receptors mediate local anti-nociceptive effects of acupuncture. *Nature Neuroscience*.

[78] Han J. S., Terenius L. (1982). Neurochemical basis of acupuncture analgesia.. *Annual Review of Pharmacology and Toxicology*.

[79] Han J. S. (1984). On the mechanism of acupuncture analgesia. *Journal of Acupuncture Research*.

[80] Han J.-S. (2004). Acupuncture and endorphins. *Neuroscience Letters*.

[81] Cheng S. S., Pomeranz B. (1981). Monoaminergic mechanism of electroacupuncture analgesia. *Brain Research*.

[82] Fei H., Xie G. X., Han J. S. (1987). Low and high frequency electroacupuncture stimulation release metenkephalin and dynorphin A in rat spinal cord. *Chinese Science Bulletin*.

[83] Han J. S., Chen X. H., Sun S. L. (1991). Effect of low- and high-frequency TENS on Met-enkephalin-Arg-Phe and dynorphin A immunoreactivity in human lumbar CSF. *PAIN*.

[84] Martens G., Laureys S., Thibaut A. (2017). Spasticity management in disorders of consciousness. *Brain Sciences*.

[85] Khan F., Amatya B., Bensmail D., Yelnik A. (2017). Non-pharmacological interventions for spasticity in adults: An overview of systematic reviews. *Annals of Physical and Rehabilitation Medicine*.

[86] Matsumoto-Miyazaki J., Asano Y., Ikegame Y., Kawasaki T., Nomura Y., Shinoda J. (2016). Acupuncture Reduces Excitability of Spinal Motor Neurons in Patients with Spastic Muscle Overactivity and Chronic Disorder of Consciousness Following Traumatic Brain Injury. *The Journal of Alternative and Complementary Medicine*.

